# Utilizing grid search cross-validation with adaptive boosting for augmenting performance of machine learning models

**DOI:** 10.7717/peerj-cs.803

**Published:** 2022-02-21

**Authors:** Muhammad Adnan, Alaa Abdul Salam Alarood, M. Irfan Uddin, Izaz ur Rehman

**Affiliations:** 1 Institute of Computing, Kohat University of Science and Technology, Kohat, Pakistan; 2 College of Computer Science and Engineering, University of Jeddah, Jeddah, Saudi Arabia; 3 Department of Computer Science, Abdul Wali Khan University, Mardan, Pakistan

**Keywords:** Cross validation, Adaptive boosting, Performance augmentation, Machine learning, Grid search

## Abstract

Corona Virus Disease 2019 (COVID-19) pandemic has increased the importance of Virtual Learning Environments (VLEs) instigating students to study from their homes. Every day a tremendous amount of data is generated when students interact with VLEs to perform different activities and access learning material. To make the generated data useful, it must be processed and managed by the proper machine learning (ML) algorithm. ML algorithms’ applications are many folds with Education Data Mining (EDM) and Learning Analytics (LA) as their major fields. ML algorithms are commonly used to process raw data to discover hidden patterns and construct a model to make future predictions, such as predicting students’ performance, dropouts, engagement, *etc*. However, in VLE, it is important to select the right and most applicable ML algorithm to give the best performance results. In this study, we aim to improve those ML and DL algorithms’ performance that give an inferior performance in terms of performance, accuracy, precision, recall, and F1 score. Several ML algorithms were applied on Open University Learning Analytics (OULA) dataset to reveal which one offers the best results in terms of performance, accuracy, precision, recall, and F1 score. Two popular ML algorithms called Decision Tree (DT) and Feed-Forward Neural Network (FFNN) provided unsatisfactory results. They were selected and experimented with various techniques such as grid search cross-validation, adaptive boosting, extreme gradient boosting, early stopping, feature engineering, and dropping inactive neurons to improve their performance scores. Moreover, we also determined the feature weights/importance in predicting the students’ study performance, leading to the design and development of the adaptive learning system. The ML techniques and the methods used in this research study can be used by instructors/administrators to optimize learning content and provide informed guidance to students, thus improving their learning experience and making it exciting and adaptive.

## Introduction

Machine Learning (ML) and Deep Learning (DL) algorithms showing good performance on a certain class of problems or datasets do not entail that they will have the same performance on other problems or datasets. If ML/DL model shows good performance results on a certain class of problems then it essentially compensates for generating degraded performance results on sets of all remaining problems (Goodfellow, Bengio & Courville, 2016). Nonetheless, in the field of education data mining, there is always some background knowledge that can be used to generate good performance results evening without knowing about the exact problem or datasets. Students learning behavior having similar features are usually classified in the same class. Often there will be outliers in the dataset with little or no noise. Rarely, there will be a time-series component attached to the dataset features. For ML/DL models, the proper dataset along with the suitable features are crucial for generating high-performance results. The choice of features influences the overall performance of the model (Loch-Olszewska & Szwabiński, 2020). Moreover, the proper ML/DL algorithm also affects the overall performance of the model as it can increase or decrease the response time of the model.

In VLEs, often a popular and stable ML/DL algorithm experiences low-performance results (Grohmann et al., 2019). For data scientists and machine learning experts, it is a challenge to reveal what causes ML/DL algorithm to generate low-performance results and what techniques can be used to improve the performance of that ML/DL algorithm (Rodrigues et al., 2019). Already, the fast spread of COVID-19 has compelled educational institutes to offers online courses to students. Various ML/DL algorithms can be used to study and comprehend the learning features of online students to provide them the needed feedback and tailored learning content. In VLEs, the performance of the ML/DL algorithm is vital to model the study behavior of students and if some ML/DL algorithms show degraded performance, then machine learning experts should come with suitable techniques to improve their performance. In this study, various ML/DL algorithms were applied to Open University Learning Analytics (OULA) dataset to predict the performance of online learners. The ML/DL algorithms showing inferior performance in terms of accuracy, precision, recall, and F1 score were selected and various techniques such as grid search cross-validation, adaptive boosting, extreme gradient boosting, early stopping, feature engineering, and dropping inactive neurons were introduced to improve their performance.

Recently, with the rapid spread of COVID-19, Virtual Learning Environment (VLE) platforms across the world experienced a massive increase in the number of online students (Dhawan, 2020). Besides, public and private sector educational institutions have also moved their courses, and learning material on the online learning platforms (Bao, 2020). Even before the COVID-19 pandemic outbreak, the explosive growth of VLEs and Massive Open Online Courses (MOOCs) has attracted millions of students where students can learn according to their needs and learning pace (Weinhardt & Sitzmann, 2019). MOOCs, VLE, Learning Management Systems (LMS), and web-based online learning systems have reduced learning costs and allow students to learn without temporal and spatial constraints. Instructors/Educators can analyze and monitor students learning behavior by evaluating their learning features stored at the computer servers. Instructors can assess students’ behavior from multiple perspectives *i.e*., predicting students’ performance, predicting students’ engagements, discovering students’ difficulties during the learning process, thereby gaining insight into students’ behavior that may cause their failure or dropout.

Contrary to traditional classroom settings, the Virtual Learning Environment (VLE) platform allows students to study from anyplace and at their own preferred time (Cofino, Atillo & Velos, 2021). In circumstances such as the COVID-19 pandemic, where social distancing is mandatory, the importance of VLE becomes more obvious and essential. The vast majority of universities are using online learning platforms such as Massive Open Online Courses (MOOCs), Learning Management System (LMS), and VLEs to provide education to those students who due to many reasons, cannot attend regular classes (Tseng et al., 2019). Popular online platforms such as Coursera, Udacity, Udemy, Edx, Khanacademy have contributed a lot in providing education of all levels to the students. VLEs allows students to further polish their skills while they are doing the job. As compare to regular class settings, where the presence of the students is mandatory, VLEs allows students to study any course at their own pace from anywhere at an affordable price. Despite having advantages, VLEs also have several challenges which educators and administrators need to consider for otherwise, VLEs may squander their effectiveness (Gillett-Swan, 2017). As there is no one to one connection between students and instructors, the form of education in VLEs is passive, where the students have to take responsibility for captivating and assisting themselves in studies. It is also noticed that in online learning settings, students after the registration process, download learning materials such as videos, notes, learning content, *etc*. and do not carry out the entire course. Due to less engagement with online courses, the total number of activities that a student must perform plunges below the recommended level. Often students’ feedback in online learning is limited, lacks face-to-face communication causing social isolation (Gillett-Swan, 2017). Therefore, the administrators of online learning must understand its disadvantages and come up with techniques that encourage students to remain active, engaged, and complete the entire course with good grades.

The overall aim of this research study is to use and compare various ML algorithms to predict students’ performance, thereby supporting instructors to perform timely intervention by recommending the suitable content to students and providing the necessary guidance. The performance of various ML algorithms is compared by using metrics such as precision, recall, f1 score, support, accuracy, macro average, and weighted average. Those ML algorithms showing unsatisfactory accuracy results are selected, and different performance improvement techniques have been applied to improve their performance. The study behavior is determined by analyzing students’ interaction with VLE platforms such as clickstream data, students’ performance in past assessments, module interest, and online content accessed. More specifically, the objectives to be addressed in this paper are as follows:

- Comparing the performance of various ML and deep learning (DL) algorithms to predict students’ performance.
- Improving the performance of those ML algorithms (DT and FFNN) that show inferior performance results in performance, accuracy, precision, recall, and F1 score.
- Using various techniques such grid search cross-validation, adaptive boosting, extreme gradient boosting, early stopping, feature engineering, and dropping inactive neurons to improve ML algorithm performance.
- Using a Decision Tree (DT) ML algorithm in determining the feature weights of students in predicting their performance.
- Classifying students according to their performance and study behavior features (Withdrawn, Fail, Pass, Distinction).
- Recommending tailored learning paths to students according to their feature weights.

The rest of the article is structured as follows: In “Literature Review” we discuss the study background and related work. “Data Description and Preprocessing” is about data description and preprocessing steps. The training of multiclass classification algorithms and their performance evaluation is discussed in “Multiclass Classification Models Training and Performance Evaluation”. The methodology is presented in “Methodology”. This study results are further discussed in “Discussion and Limitations”. The study conclusion and future work is discussed in “Conclusion and Future Work”.

## Literature Review

Table 1 presents a comparison of various studies carried out on VLE and MOOC datasets, their objectives, ML algorithms used, and evaluation metrics with the availability of data mining tools, researchers are experimenting on various educational datasets to know about students’ study behavior, factors affecting their performance, and attrition (Gupta & Sabitha, 2019). The results generated from the learning analytics dataset helps instructors in decision-making and supporting students. Predicting students’ performance based on their feature weights is a new phenomenon in the educational domain, helping instructors to know about students learning behavior in detail and to provide timely corrective strategies to avoid students from course dropout. In literature, there is substantial debate over determining factors affecting students’ behavior, avoiding students dropout, and providing timely support in VLE. Numerous studies had been carried out to determine the features contributing to students’ study performance. Features such as study time, study duration, online engagement rate, type of learning content, background knowledge, earlier education, parental education, student-instructor interactivity, course prerequisite, motivational and social factors, assessment performance are directly associated with the students’ final performance in VLE.

**Table 1:** Comparative Analysis of studies using ML techniques on VLE and MOOC datasets.

Authors	Objective	ML model	Performance & Evaluation
Helal et al. (2018)	Academic performance prediction by considering student heterogeneity	Naïve Bayes, J48, SMO JRip	Naïve Bayes with best Accuracy = 85%
Heuer & Breiter (2018)	Using daily online activity to predict students’ success	Decision Tree, Random Forest, Logistic regression, Support Vector Machine	SVM with f1 score = 91% and accuracy = 87% precision = 93% recall = 89%
Herodotou et al. (2019)	Using predictive learning analytics to empower online teachers	Chi-square Kruskal-Wallis H test	55.9%, *p* = 0.0003 for high group students 48.1%, *p* = 0.0006 for low group students
Okubo et al. (2017)	Using recurrent neural network to predict students performance in multiple courses	RNN	Accuracy = 84.6%
Waheed et al. (2020)	Deploying deep learning model to predict students performance	Deep Artificial Neural Network	Accuracy: DANN = 84–93%
			Logistic Regression = 79.82–85.60%
			Support Vector Machine = 79.95–89.14%
Manrique et al. (2019)	Using classification algorithms to predict students dropout	Naïve Bayes (NB)	Precision, Recall, Accuracy, f1 Random Forest Accuracy = 86%, Precision = 86%, f1 = 85% GBT Recall = 86%
		Support Vector Machine (SVM)	
		Random Forest (RF)	
		Gradient Boosting Tree (GBT)	
Cobos & Olmos (2018)	Predicting students dropout and success in MOOC for professional learning	Boosted Logistic Regression	Stochastic Gradient Boosting
		Stochastic Gradient Boosting	Neuronal Network
		Random Forest, Naïve Bayes	Random Forest with accuracy above 80%
		Neuronal Network	
		Support Vector Machine	
		K-Nearest Neighbors	
		Classification Tree	
		Extreme Gradient Boosting	
Jha, Ghergulescu & Moldovan (2019)	Predicting dropout and performance using ensemble, regression and deep learning technique	Generalized Linear Model	Deep learning with 94% accuracy for dropout prediction.
		Gradient Boosting Machine	Deep learning with 96% for performance prediction
		Distributed Random Forest	
		Deep Learning	
Xing & Du (2019)	Intervening students to avoid dropout by leveraging deep learning	K-Nearest Neighbors	Deep Learning with 98% accuracy
		Support Vector Machine	
		Decision Tree Deep Learning	
Youssef et al. (2019)	Predicting learners’ dropout in MOOC based on efficient algorithm and feature selection	Support Vector Machine	Random Forest with 97% accuracy using RFE feature selection method
		K-Nearest Neighbors	
		Decision Tree, Naïve Bayes	
		Logistic Regression	
		Random Forest	
Hmedna, El Mezouary & Baz (2019)	Identification of learning styles using predictive model	Neural Networks, Decision Tree	Decision Tree with the highest accuracy of 99%, Precision = 99%, Recall = 99%, f1 score = 99%, micro-precision = 99%, macro-precision = 98%
		Random Forest	
		K-Nearest Neighbors	
Yu, Wu & Liu (2019)	Predicting students performance in MOOC with clickstreams data	K-Nearest Neighbors	ANN with the highest accuracy of 96%
		Artificial Neural Networks	
		Support Vector Machines	
Imran, Dalipi & Kastrati (2019)	Evaluating the performance of deep neural networks to predict students dropout in MOOC	Deep Neural Network (DNN)	DNN with 99% accuracy when using 64 neurons
Ding et al. (2019)	Improving the performance of predictive models by effective feature selection using unsupervised machine learning	Long Short-Term Memory (LSTM)	LSTM with best feature selection
		Convolutional Neural Networks (CNN)	

Damaševičius (2009) analyzed the application of association rule mining for evaluating the students’ academic results and improving course material. A novel metric was introduced called cumulative interestingness for assessing the strength of association rule to determine the factors influencing examination results, important course topics, and students’ study behavior. The strength of the proposed framework was validated by carrying out a case study in an informatics course. The research study demonstrated that the association rule mining technique can help both instructors and students in improving the learning content and knowing which course topic caused the most difficulties for the students during their study. The major drawback of this study was that no topic map was provided to the students that would help them in selecting which topic to study first.

Aydoğdu (2020) predicted the performance of 3,518 students, who were actively participating in LMS activities using Artificial Neural Networks (ANN). The features selected for performance prediction were gender, assessment score, study duration, number of logins made, attendance in the live session, homework score, attendance in archived courses, and time spent on archived courses. The ANN was able to make predictions with 80.47% accuracy with attendance in the live session, attendance in archived courses and study time duration being the most significant features contributing to the prediction of the final performance.

Hew et al. (2020) reasoned that the success of MOOCs is not associated with students’ course completion rate, but as the extent of students’ contentment with the course. Students’ satisfaction with MOOCs can enable educational institutions to extend their reach to more people and use MOOCs as a source of revenue. Their study adopted a supervised ML algorithm, hierarchical linear modeling, and sentiment analysis to analyze the 249 MOOC course features. A total of 6,393 randomly selected students’ data was also added to analyze the students’ perception of MOOCs. The results revealed that features such as course instructor, assessment score, course content, and timetable play a considerable role in justifying students’ contentment with MOOC while course major, structure, duration, workload, content type (video, text, *etc*.), MOOC interaction, and perceived difficulty play a less significant role.

Hlioui, Aloui & Gargouri (2018) argued that one of the important factors in reducing dropout rates in VLE is the accurate and timely prediction of students’ engagement level and providing tailored assistance. They proposed a survival modeling technique to analyze various feature roles on attrition in Open University, UK. The learning outcome was perceived from a student engagement perspective, which asserts that the more a student is engaged with VLE, the better the knowledge and skill acquisition is. To enhance students’ efficiency, the course instructor was provided with an innovative process where the instructor can interfere with a weak student during the learning process by using dialog prompts and providing the needed learning material.

With the emergence of MOOCs platforms and ML techniques in the past decade, studies on analyzing and predicting VLE students’ performance, dropouts, engagement, and difficulties, which allows for instructors timely intervention to support students, are not rare. The easiest way to predict students’ online learning behavior is to get data from log records of MOOCs and LMS and analyze it (Helal et al., 2018; Jiang et al., 2014; Romero et al., 2013; Xing et al., 2016).

However, researchers may face difficulties in the data collection process, as MOOCs and LMS are not available for each subject in every university. Moreover, the online systems store data about the offered courses, and often do not consider storing students’ behavioral features for all courses. To overcome data collection difficulties, researchers often use an online questionnaire survey as an effective tool to collect data about students’ academics achievements and study behavior (Apuke & Iyendo, 2018). On the other hand, an online questionnaire study often suffers from data credibility and assurance issues as the data may be inaccurately reported by the students (Giunchiglia et al., 2018; Lee et al., 2017). To accurately predict students’ performance grades, it is essential to find out the key features of online platform usage that could be used as input variables by ML models for contributing to effective prediction.

**Figure 1:**
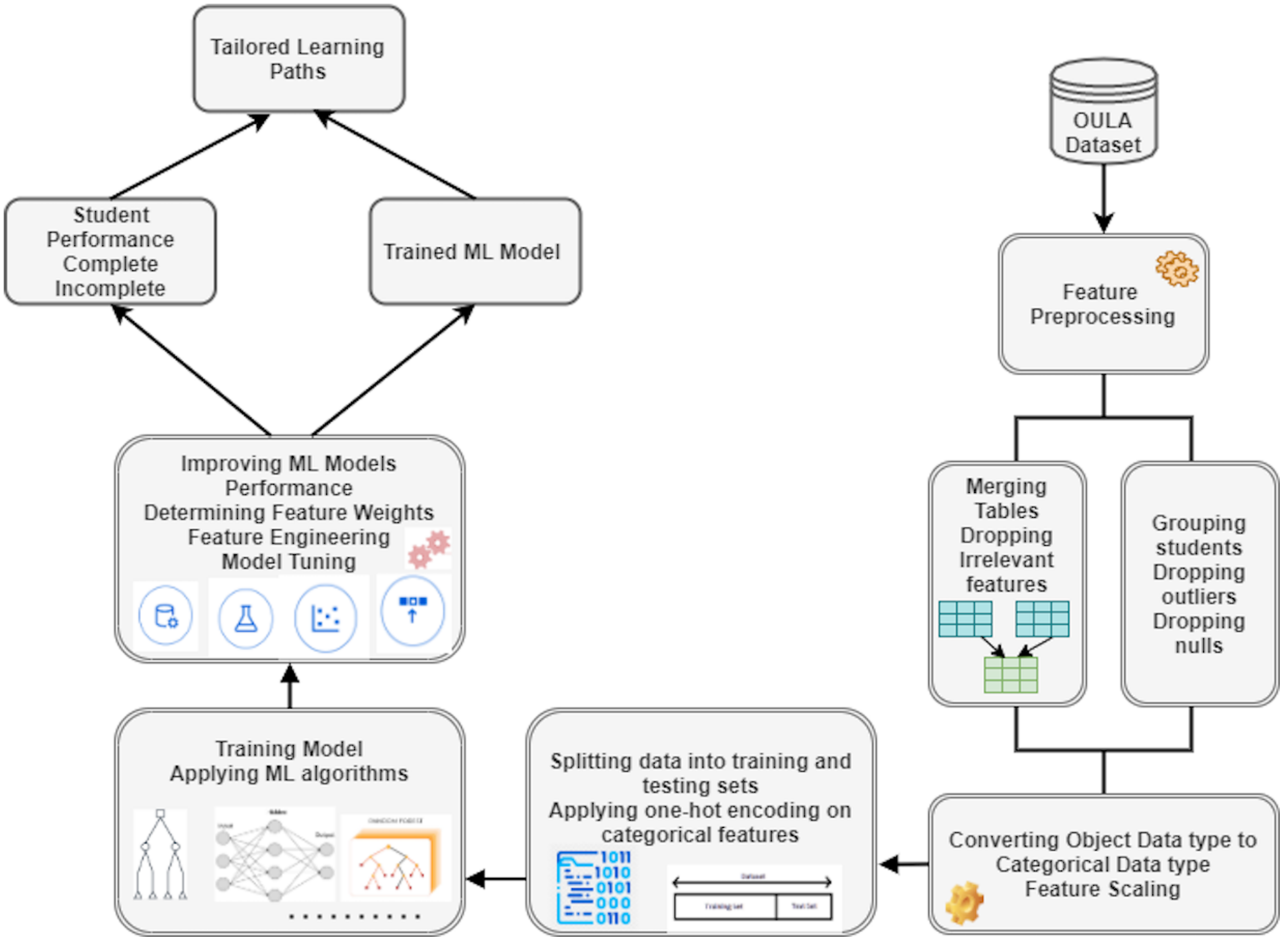
Workflow of the proposed machine learning based VLE architecture.

In this study, various ML algorithms were employed to determine which algorithm gives the best results in terms of predicting students’ performance in different course modules. After merging tables and selecting the most relevant features, MIN-MAX scaling technique was used to normalize the data. Before feeding features into various ML algorithms, a train-test split was performed with 85% data reserved for training and 15% reserved for testing the models. Figure 1 presents the working of the proposed ML based VLE architecture.

## Data Description and Preprocessing

We used Open University Learning Analytics (OULA) dataset that includes information about the students’ demographics, course registration, VLE interactions, clickstreams, assessments, and final performance scores for training ML models. The dataset is available freely at Open University Learning Analytics Dataset and is officially certified by Open Data Institute (ODI): http://theodi.org/.

The students, courses, registration, and clickstream data in the acquired OULA dataset are spread across seven tables, with students as the focal point of information. For this study, 32,593 students’ data was gathered for 9 months. The students’ final performance is categorized into four classes/grades with 31% withdrawn students, 22% fail students, 38% pass students, and 9% of students who secured distinction position. Seven courses known as modules were offered to students, with each module delivered at least twice a different time in a year. The procured raw data having seven tables are connected with identifier columns. The tables contain information about assessments, assessment types, assessment submission dates, courses, VLE resources types, students’ interaction with VLE in the form of clickstreams, course registration information, and student demographics. The VLE clickstream consist of information about various activities carried out by students on heterogeneous modules such as discussion forums, accessing course materials, students’ logins, visiting OU main page, subpage, URL, and glossary, *etc*. Their clickstream sum records the interest of the students in each activity type.

### Processing the raw dataset

The OULA features in seven tables were combined into one table having independent and dependent features. For this research study, we selected students’ final result as the dependent or target feature, whereas the rest of the features were selected as the predictors. First, *Students Assessments* and *Assessment* tables were merged into the *Combine Assessment* table to know to which module each assessment belongs to. Several unnecessary features such as code_presentation, assessment_type, date, weight from the *Assessment* table, and is_banked, date_submitted from the *Students Assessments* table were dropped before the merge operation as we only needed code_mudule and students score features. The features included in the *Combined Assessments* table included id_assessment, id_student, code_module, and assessment score features. In the second step, the *Combine Assessments* was merged with the *Student Info* table to include students’ assessment scores in each code module with the *Student Info* table. The resultant *Student Assessment Info* table was further merged with the *VLE Grouped* table on id_student and code_module features using Python left join operation. The *VLE Grouped* table was created by merging Student VLE and VLE table and dropping unnecessary features. The features of the *VLE Grouped* table were grouped by id_student and code_module features. The *VLE Grouped* table presents students registered in each code module and their clickstream sum in that code module. The *Final Info* table contains all important information about student assessments, code_modules, VLE interaction data, number of previous attempts, studied credits, *etc*.

#### Dropping null values

The *Final Info* table consists of 14 features with 30,358 feature instances. All feature instances with null, N/A, or missing data were dropped as ML algorithms can’t use them during their training process. Invalid or missing instances usually trigger an ML algorithm to produce misleading outcomes or less accurate results. After dropping the missing values, the *Final Info* table consisted of 28,939 feature instances.

#### Converting object data types features to categorical features

It was revealed that several features data type was changed to object data type during tables merge operation. These features were converted to categorical data type due to their values characteristics. These features included code_module, code_presentation, gender, region, highest_education, imd_band, age_band, disability, and final_result. The categorical features allow ML algorithms to use memory efficiently and leads to accurate ML model results.

#### Scaling features

We noted that some features were having variation in their magnitude and range, such as sum_click, num_of_prev_attempts, and studied_credits. One independent feature may gain higher weightage over other features by the ML model when not properly scaled. The three features, as mentioned above, were scaled to one unit using the standard scalar as:

(1) $$z = (x - \mu )/\sigma$$

where x is the feature, $\mu$ is the feature mean, and $\sigma$ is the feature standard deviation. Feature scaling helps the ML model in speeding up the training process and generating accurate results.

#### Performing one-hot encoding

Before fitting the data into the ML model, a one-hot encoding technique was applied to all categorical features. Converting categorical features to one-hot encoding is important, as most ML algorithms cannot work with them directly. They require all independent categorical features and dependent categorical features to be converted into a numeric data type. One-hot encoding leads to an efficient implementation of ML algorithms and is also necessary for ML algorithms parallel operations. After performing the one-hot encoding technique on all the categorical features, we get a dataset having 50 input features and 28,939 feature instances. The dependent feature called final_result was also converted, and four feature columns were generated, one for each performance class, as shown in Table 2.

**Table 2:** Performing one-hot encoding of the final result feature.

Withdrawn	Fail	Pass	Distinction
1	0	0	0
0	1	0	0
0	0	1	0
0	0	0	1

## Multiclass Classification Models Training and Performance Evaluation

After the data preprocessing and exploratory steps, 11 ML classifiers and one Deep Learning (DL) classifier were used to train the models and predict students’ final performance. The 11 ML classifiers included Random Forest (criterion = ‘gini’), Random Forest (criterion = ‘entropy’), AdaBoost, Extra Tree classifier, K-Nearest Neighbor, Decision Tree, Support Vector Machine (SVM), Gradient Boosting, Logistic Regression, Gaussian Naïve Bayes, and Bernoulli Naïve Bayes classifier. The one DL classifier included Feed Forward Neural Network (FFNN). For each of these classifiers, different experiments were carried out with different configuration parameters; however, the models having optimized results were selected. The models were selected based on accuracy, precision, recall, f1, support, macro average, and weighted average score. Tables 3–5 present ML classifiers with inferior, average, and superior performance scores. It can be observed that the classification models trained with gradient boosting, extra tree classifier, random forest ‘criterion = gini’, and random forest ‘criterion = entropy’ classifiers have the highest performance score. For brevity, we selected FFNN and Decision Tree classifiers for tuning and for further improving their prediction performance scores using various techniques.

**Table 3:** ML classifiers with inferior performance scores.

Gaussian NB	Precision	Recall	f1-score	Support
Distinction	0.32	0.48	0.38	464
Fail	0.35	0.37	0.36	927
Pass	0.62	0.46	0.53	2,067
Withdrawn	0.40	0.51	0.45	883
Accuracy			0.45	4,341
Macro avg	0.42	0.46	0.43	4,341
Weighted avg	0.49	0.45	0.46	4,341
SVM				
Distinction	0.52	0.17	0.26	519
Fail	0.46	0.11	0.18	928
Pass	0.64	0.55	0.59	2,000
Withdrawn	0.32	0.81	0.46	894
Accuracy			0.46	4,341
Macro avg	0.49	0.41	0.37	4,341
Weighted avg	0.52	0.46	0.44	4,341
Bernoulli NB				
Distinction	0.34	0.08	0.13	464
Fail	0.37	0.24	0.29	927
Pass	0.56	0.77	0.65	2,067
Withdrawn	0.41	0.38	0.39	883
Accuracy			0.50	4,341
Macro avg	0.42	0.37	0.37	4,341
Weighted avg	0.47	0.50	0.47	4,341
KNN				
Distinction	0.46	0.49	0.48	464
Fail	0.36	0.41	0.38	927
Pass	0.61	0.67	0.64	2,067
Withdrawn	0.47	0.27	0.34	883
Accuracy			0.52	4,341
Macro avg	0.48	0.46	0.46	4,341
Weighted avg	0.51	0.52	0.51	4,341

**Table 4:** ML Classifiers with average performance score.

Decision tree	Precision	Recall	f1-score	Support
Distinction	0.42	0.44	0.43	464
Fail	0.40	0.42	0.41	927
Pass	0.66	0.63	0.64	2,067
Withdrawn	0.46	0.47	0.46	883
Accuracy			0.53	4,341
Macro avg	0.49	0.49	0.49	4,341
Weighted avg	0.54	0.53	0.53	4,341
Logistic				
Regression				
Distinction	0.67	0.32	0.43	519
Fail	0.48	0.31	0.38	928
Pass	0.63	0.85	0.72	2,000
Withdrawn	0.54	0.47	0.50	894
Accuracy			0.59	4,341
Macro avg	0.58	0.49	0.51	4,341
Weighted avg	0.58	0.59	0.57	4,341
Ada boost				
Distinction	0.52	0.52	0.52	464
Fail	0.46	0.40	0.43	927
Pass	0.67	0.79	0.73	2,067
Withdrawn	0.55	0.40	0.46	883
Accuracy			0.60	4,341
Macro avg	0.55	0.53	0.53	4,341
Weighted avg	0.58	0.60	0.59	4,341
FFNN				
Distinction	0.57	0.51	0.54	471
Fail	0.50	0.44	0.47	946
Pass	0.67	0.83	0.74	2,036
Withdrawn	0.59	0.38	0.46	888
Accuracy			0.62	4,341
Macro avg	0.58	0.54	0.55	4,341
Weighted avg	0.61	0.62	0.60	4,341

**Table 5:** ML classifier with superior performance score.

Gradient boosting	Precision	Recall	f1-score	Support
Distinction	0.68	0.46	0.55	519
Fail	0.51	0.38	0.43	928
Pass	0.66	0.86	0.75	2,000
Withdrawn	0.61	0.47	0.53	894
Accuracy			0.63	4,341
Macro avg	0.61	0.54	0.56	4,341
Weighted avg	0.62	0.63	0.61	4,341
Extra tree classifier				
Distinction	0.65	0.43	0.52	464
Fail	0.51	0.40	0.45	927
Pass	0.67	0.85	0.75	2,067
Withdrawn	0.60	0.47	0.53	883
Accuracy			0.63	4,341
Macro avg	0.61	0.54	0.56	4,341
Weighted avg	0.62	0.63	0.62	4,341
Random forest ‘gini’				
Distinction	0.66	0.48	0.56	464
Fail	0.56	0.45	0.50	927
Pass	0.69	0.86	0.77	2,067
Withdrawn	0.64	0.50	0.57	883
Accuracy			0.66	4,341
Macro avg	0.64	0.57	0.60	4,341
Weighted avg	0.65	0.66	0.65	4,341
Random forest ‘entropy’				
Distinction	0.66	0.48	0.56	464
Fail	0.55	0.44	0.49	927
Pass	0.69	0.87	0.77	2,067
Withdrawn	0.65	0.48	0.56	883
Accuracy			0.66	4,341
Macro avg	0.64	0.57	0.59	4,341
Weighted avg	0.65	0.66	0.64	4,341

## Methodology

The performance of ML algorithms varies on the nature of problems and datasets. We could not assume that if 1 ML algorithm performs well on a certain class of problems, it will also perform well on the rest of the problems. Often, in data science, ML experts are surprised by a degraded ML algorithm performance on new and different problems. Therefore, it is important to evaluate various ML algorithms on one problem and to determine which one performs best based on their output results. Besides the aforementioned facts, luckily, there is always some background knowledge even without knowing anything about the datasets such as; (1) in multiclass classification problems, samples having similar features are classified in a common class, (2) it is likely that there will be some outliers in the dataset, (3) there is going to be little noise in the current dataset, (4) which ML algorithms are used commonly for multiclass classification problems, (5) which algorithms are considered to have the excellent running time, *etc*. We first selected the Decision Tree (DT) ML algorithm for students’ performance prediction as it is considered relatively robust in multiclass classification problems. The performance of the DT model was further improved using various techniques as increasing model performance where it has shown unsatisfactory result is one of the objective of this study.

### Decision tree (DT)

**Figure 2:**
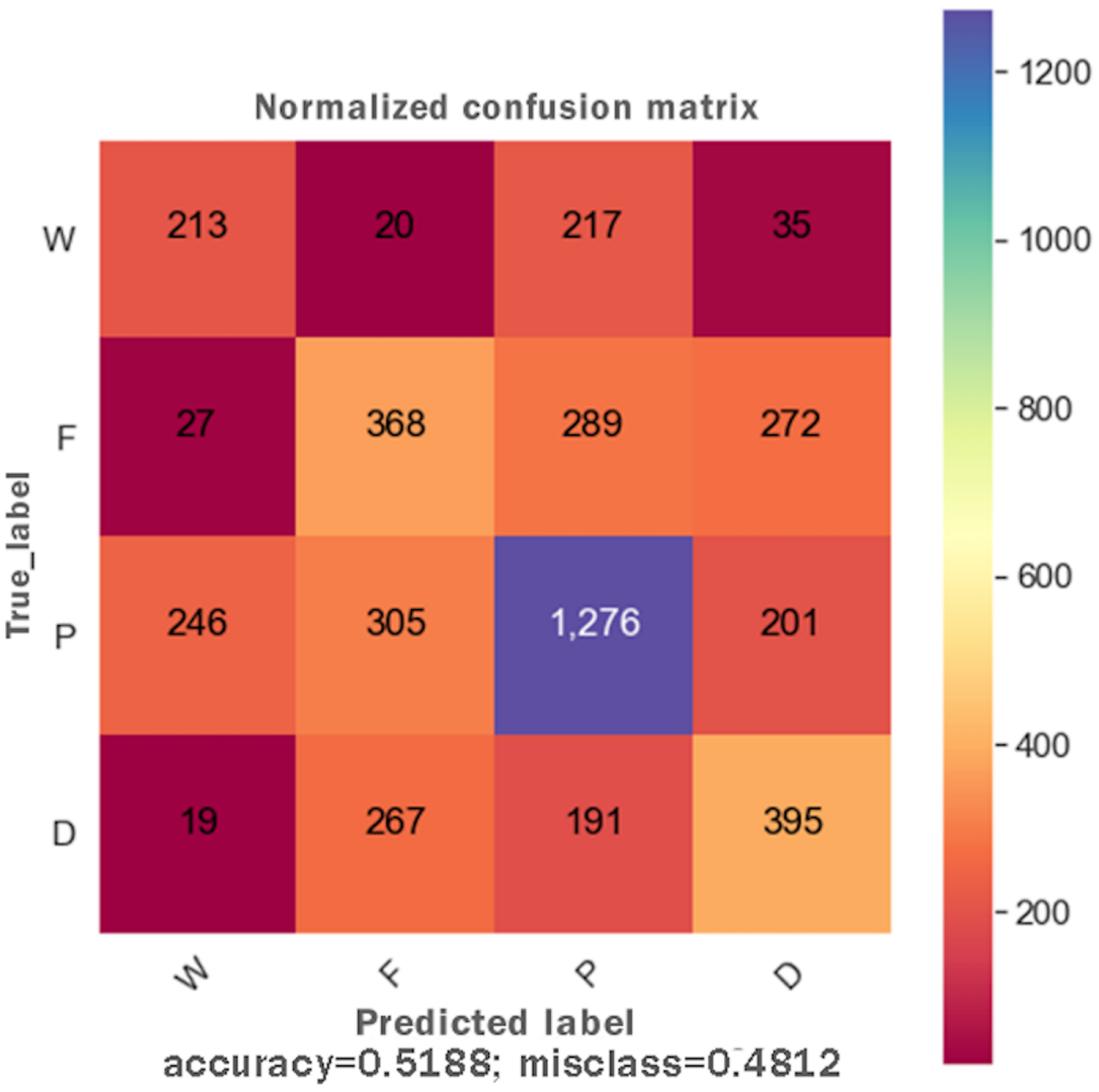
Confusion matrix for the DT model.

**Figure 3:**
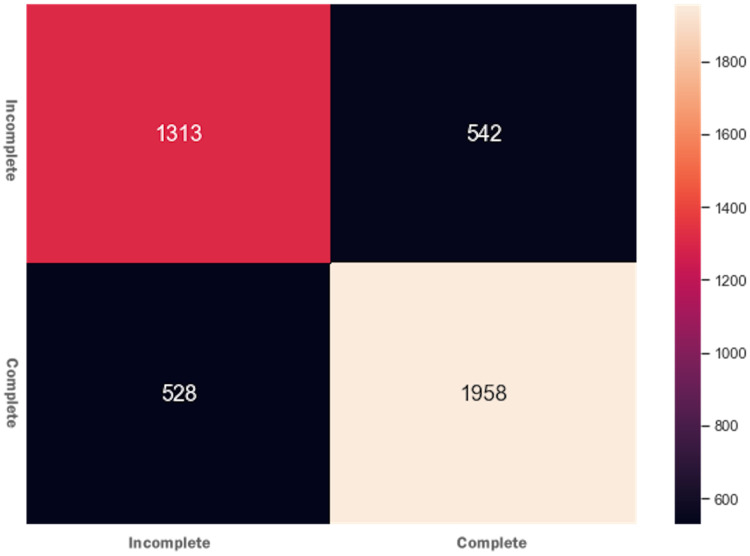
Confusion matrix for the DT model after feature engineering.

DTs are adaptable to multiclass classification problems, regression settings, and continuous data. DTs have leaf nodes, intermediate nodes, and one root node. A split is made on each node by selecting a feature value. All samples having similar feature value go in one branch, whereas all other goes to the other branch. Gini and information gain are the most common splitting metrics used in building a DT. The general rule for splitting metrics is based on feature values giving the greatest separation for the predictor features. For training the DT model, the *final result* feature was set as a dependent feature, whereas all other features were set as independent features. Figure 2 shows the DT model classifier’s confusion matrix after the data was fitted and the model was trained/tested. The results were not satisfactory as a low score for accuracy = 0.518, and f1 = 0.517 were achieved. The resultant confusion matrix shows the true label on the Y-axis, whereas the predicted labels are displayed on the X-axis. The diagonal numbers correctly predicted final result labels, whereas the rest of the labels are incorrectly predicted. It can be noticed that the highest numbers of the correctly predicted label are *Pass* while the lowest numbers of the correctly predicted label are *Withdrawn*. One reason for low accuracy and f*1* score could be the class imbalance problem because the Withdrawn (10,156), Fail (7,052), Pass (12,361), and Distinction (3,024) classes are not distributed evenly. To increase DT prediction, accuracy, and *f1* score, *Withdrawn* and *Fail* classes were merged into *incomplete* class while *Pass* and *Distinction* classes were merged into *complete* class. We once again fitted and executed the DT model classifier and received higher accuracy = 0.753 and f1 = 0.753 score. Figure 3 shows the confusion matrix for the DT model classifier for complete/incomplete classes. The result shows that after performing merge operation on final result labels, we could increase the accuracy score by almost 24%. To further improve the DT model classifier’s prediction accuracy, we used a technique called Grid Search Cross-Validation.

### Improving DT model classifier performance using grid search cross-validation

Grid Search Cross-Validation (GSCV) is a technique used to optimize hyper-parameters. It finds the best combination of hyper-parameters that give optimal results for the model performance. For example, during the model training process, GSCV creates multiple models, each with a unique combination of hyper-parameters. The goal of GSCV is to train each of these models and evaluate their performance using cross-validation. Ultimately, the model that gives the best results is selected. The DT model classifier was once again fitted with the following hyper-parameters. Values for maximum depth = [2, 3, 4, 5, 6, 7, 8, 9, 10], values for maximum leaf nodes = [8, 18, 28, 38, 48, 59, 69, 79, 89, 100], nodes splitting criterion = [‘gini’, ‘entropy’], class_weight = [‘balanced’, None], cross-validation = 3. The GSCV generated the following best hyper-parameters for the DT model classifier with an accuracy score of 0.83. ‘class_weight’ = None, ‘criterion’ = ‘gini’, ‘max_depth’ = 7, ‘max_leaf_nodes’ = 38. Using the GSCV technique we were able to increase the performance score of the DT model by 9%.

### Using ensembling to increase DT model classifier performance

Ensembling is a technique that combines several models into one bigger model, which produces better results than any of its constituents. Ensembling techniques usually take a longer time in ML model training, but even a simple model trained using ensemble technique can outperform more advanced and specialized models. Popular and common ML ensemble techniques include Bagging, Boosting, Random Forest, Extra-Trees, AdaBoost, Gradient Boosting Machine (GBM), and XGBoost. For brevity, we used two ensemble techniques, *i.e*., AdaBoost and XGBoost.

#### Adaptive boosting (AdaBoost)

**Figure 4:**
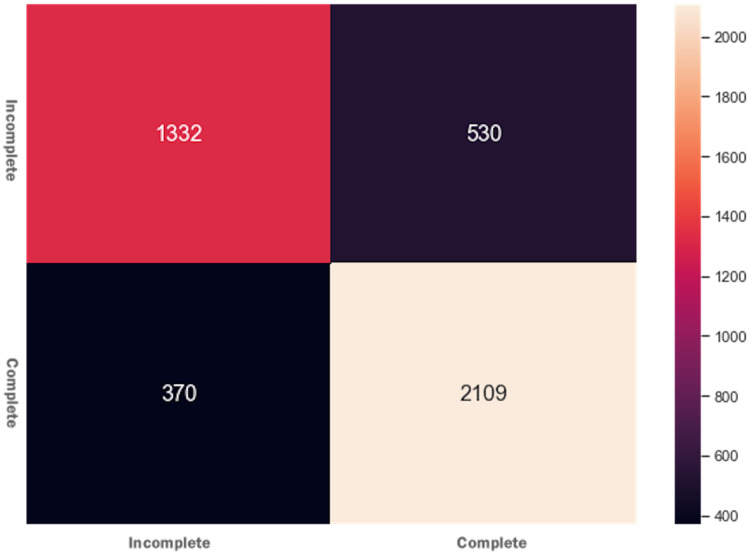
Confusion matrix for the AdaBoost ensembling technique.

The AdaBoost ensembling technique’s basic idea is that it combines several weak learning models into a strong learning model, and the predictors’ features are trained sequentially. It gives more weights to those features’ instances that previously were responsible for model underfitting. The training and testing features were fitted to the AdaBoost classifier and received accuracy = 0.792 and f1 = 0.791. The results determine that the AdaBoost classifier could not improve accuracy and the f1 score of the DT model. Figure 4 presents the confusion matrix for AdaBoost ensembling technique.

#### eXtreme gradient boosting (XGBoost)

**Figure 5:**
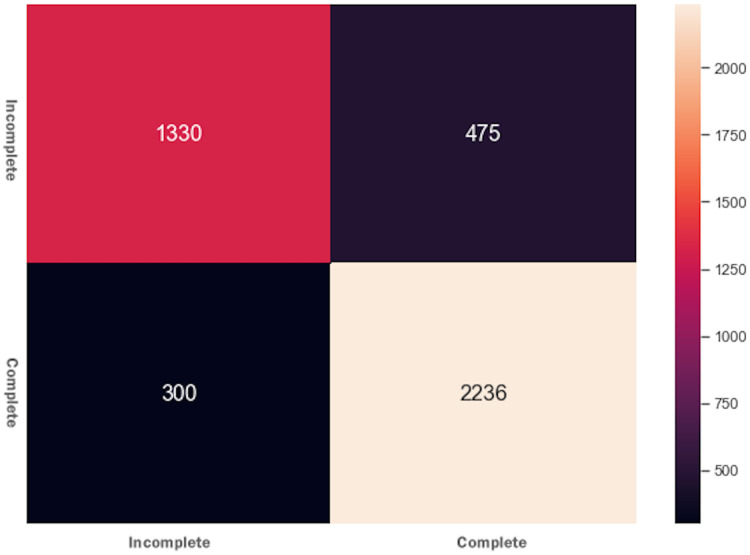
Confusion matrix for the XGBoost algorithm.

XGBoost is the cutting edge and one of the most advanced supervised ML algorithms. As compared to other ensemble classifiers, it is comparatively fast, parallelizable, gives better performance scores, and has a variety of tunning parameters for regularization, cross-validation, missing values, *etc*. After fitting the data and running the model, we received an accuracy of 0.82 and an f1 score of 0.81. The XGBoost classifier results were very close to the GSCV technique, and there was no improvement in model performance. The confusion matrix for XGBoost is shown in Fig. 5 where it can be noticed that XGBoost accurately predicted more elements than the AdaBoost algorithm.

**Figure 6:**
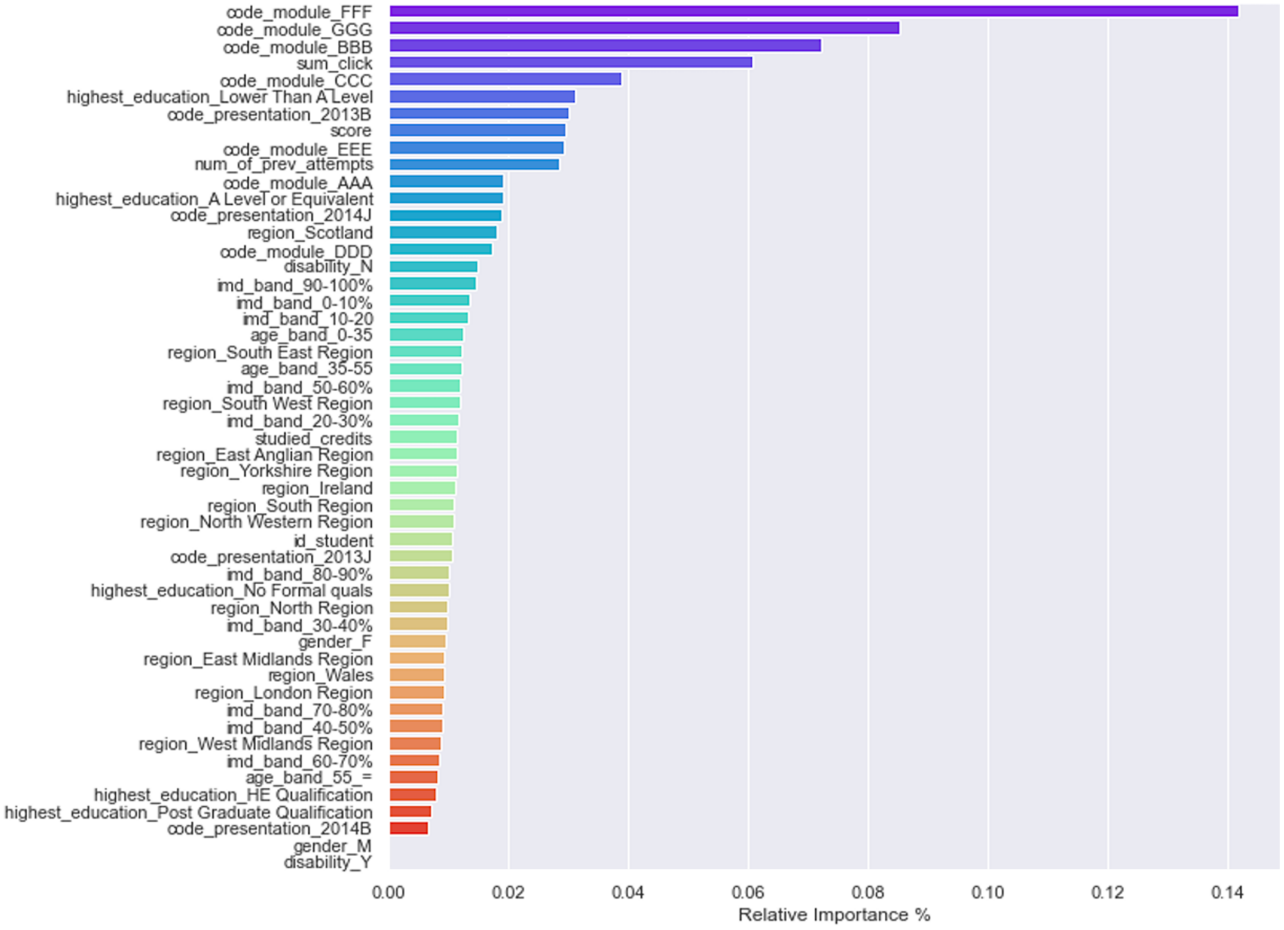
Features importance/weights in predicting the final performance.

One of the benefits of tree-based classification algorithms is that they are easily interpretable and explainable, leading to easy calculation of factors such as features’ importance and decision paths. In VLE, it is essential to determine each feature’s relative weights in predicting students’ performance. Feature weights can tell instructors about students’ strengths and weaknesses and how to provide them adaptive guidance. By looking at feature weights, the LMS or VLE platforms can recommend adaptive paths to students, making the learning process easy and adaptive. Most MOOCs/VLE and LMS follow the one-size-fits-all approach where the same learning content is delivered to all students regardless of their learning pace and performance. Contemporary online systems assign equal weights to all students’ features while providing them help and guidance. Not considering feature weights is the main reason for students’ misclassification in online learning platforms. Thus, due to students’ misclassification, they do not get adaptive and tailored learning content. XGBoost classifier was once again used to determine each feature’s importance in predicting students’ final results. Figure 6 shows a bar plot displaying the relative weights of independent features in predicting the final result feature. The top 10 features that significantly contributed to leveraging the final performance of students were: module FFF, module GGG, module BBB, sum_clicks, module CCC, higher education lower than A level, code_presentation_2013B, assessment scores, module EEE, and the number of previous attempts. Apart from modules and code_presentation features, we noticed that the number of clicks, assessment scores, and previous attempts significantly increase students’ final performance.

#### Assessing XGBoost model with learning curves

A good ML model generalizes well over unseen feature samples. Even if the inputs are new, a good ML model gives a sensible output. The application and performance of a model depend heavily on the generalization of the model. If the model generalizes well, it will serve its objectives; otherwise, it will show poor performance and results. Underfitting and overfitting are two common deficiencies in ML models that halt them from generalizing on unseen data. Overfitting occurs when a model learns a lot during the learning stage, and the overall cost is minimal, but the model performs poorly on unseen data. This phenomenon causes a model to be unreliable, and it does not generalize well.

On the other hand, underfitting occurs when a model has not learned enough during its training stage. A model suffering from underfitting shows poor performance and low generalization on testing and unseen data. Specifically, suppose a model does not predict well. It is highly biased and suffers from underfitting, whereas if a model is sensitive to slight variation between feature samples, it has high variance and suffers from overfitting. Model performance that lies between underfitting and overfitting is desirable and generalizes well. A good supervised model always has an intrinsic bias-variance tradeoff during its training stage. Generally, this is possible by tuning the model’s hyperparameters, such as with the maximum depth of a DT.

**Figure 7:**
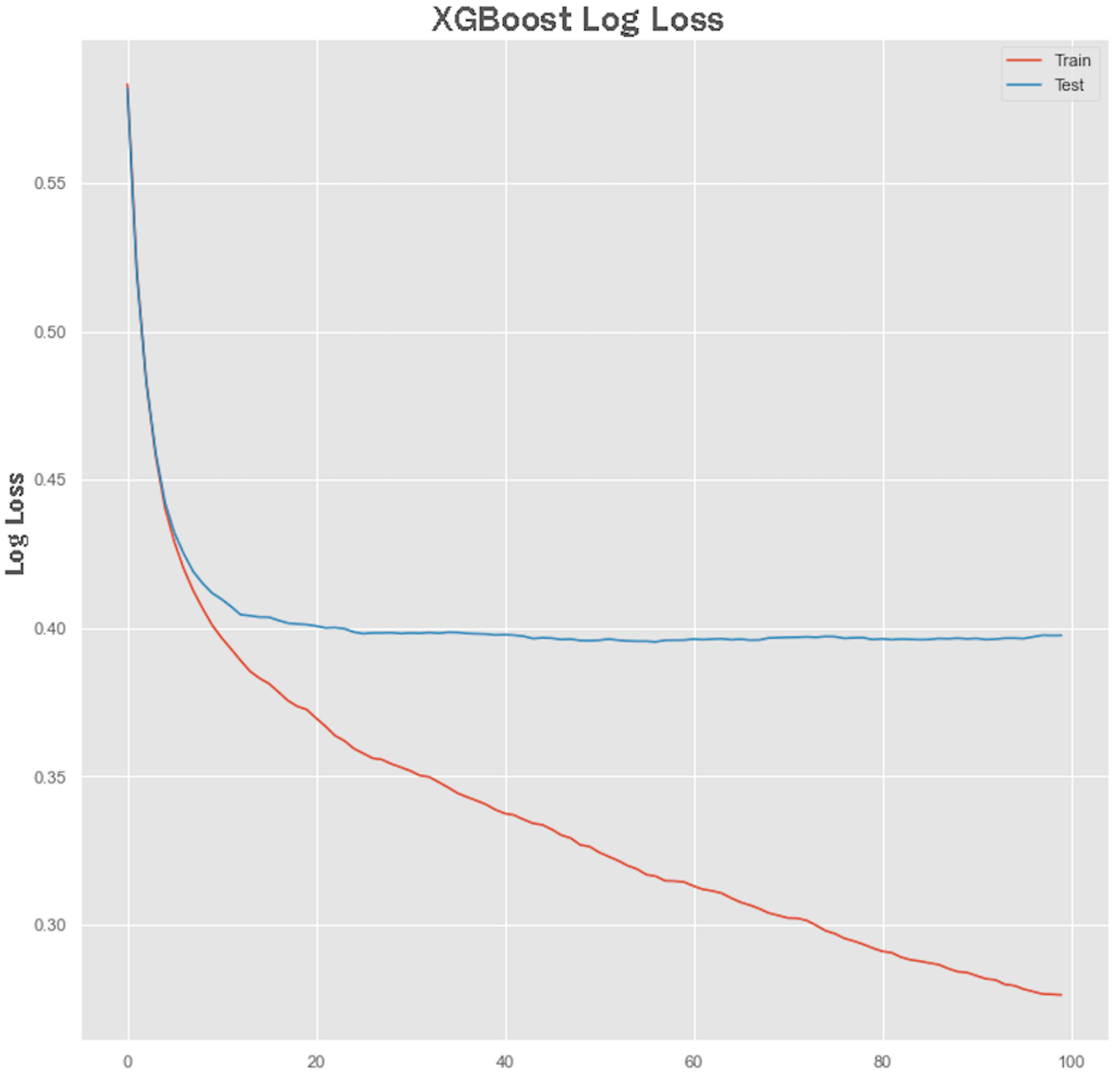
XGBoost log loss performance.

**Figure 8:**
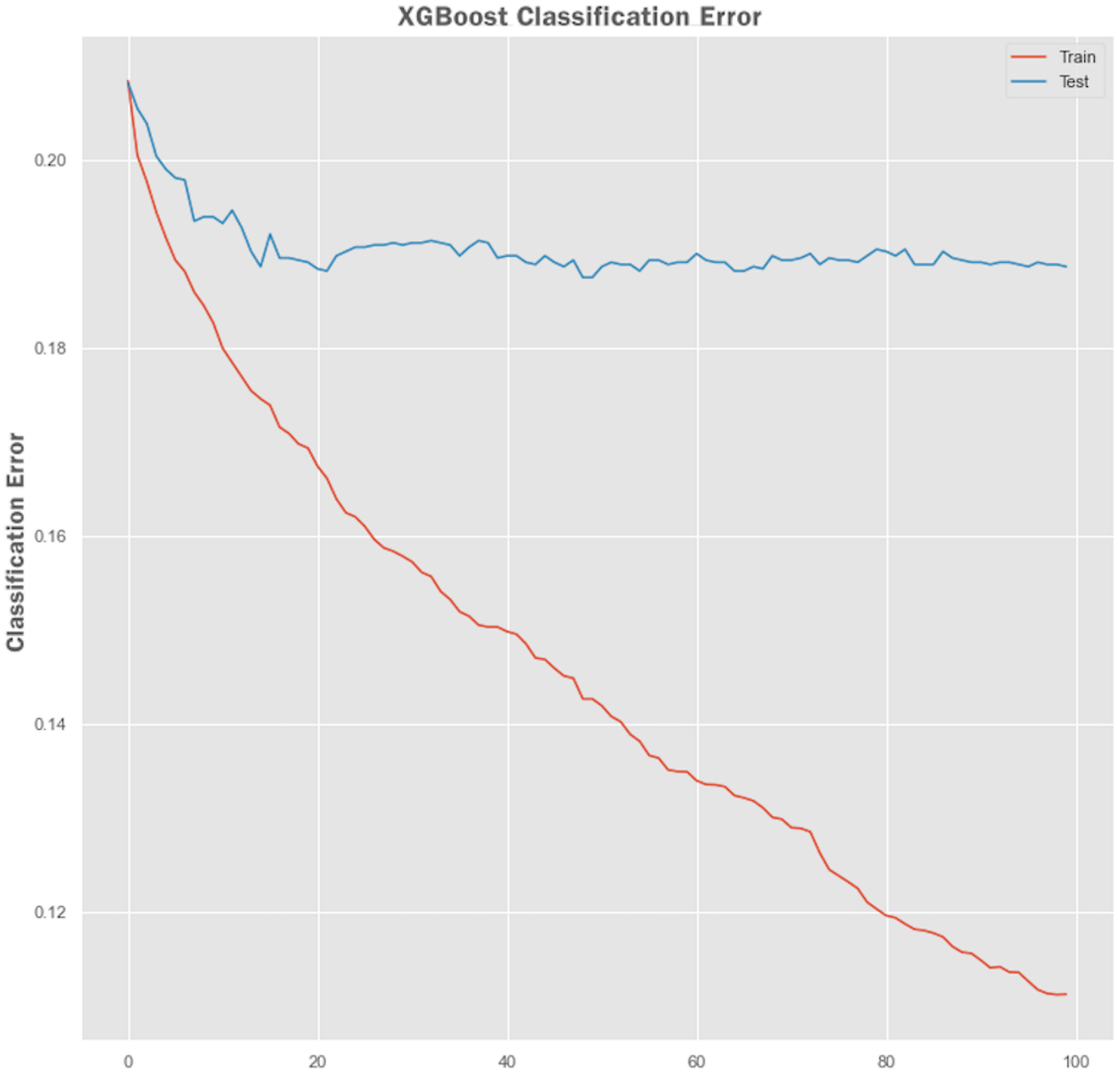
XGBoost classification error performance.

Non-linear algorithms such as XGBoost frequently suffer from overfitting problems. A technique called early stopping was used during the XGBoost training stage to avoid it from overfitting. Before applying the early stopping technique, we plot a learning curve to retrieve the performance of the XGBoost model both on training and testing sets. The performance results of the XGBoost model are shown in Figs. 7 and 8 showing the logarithmic loss and classification error of the XGBoost model. Looking at the logarithmic loss plot in Fig. 7, it looks like there is a chance to halt the learning early, feasibly somewhere across epoch 0 to epoch 20. A similar observation was made for the classification error plot in Fig. 8 where it appears to stop the training process at epoch 10.

#### Early stopping with XGBoost model training

We configured XGBoost to stop after certain numbers of epoch, after which no improvement in the model performance was observed. The parameters specified for early stopping were early_stopping_rounds = 10, eval_metric = “logloss”, eval_set = eval_set. The XGBoost model stopped training at epoch 41, and at epoch 31, the best model loss was observed with an accuracy of 80.83%. We can innate bias-variance tradeoff through model early stopping during the training process, thus avoiding the model to suffer from underfitting and overfitting problems.

### Feed-forward neural networks (FFNN)

As compared to normal neural networks, DFFNN can extract features from low-level details, find hidden patterns and feature ranks. Established on old feature instances, DFFNN can predict unseen data outcomes, and the prediction accuracy improves with newly obtainable data.

**Figure 9:**
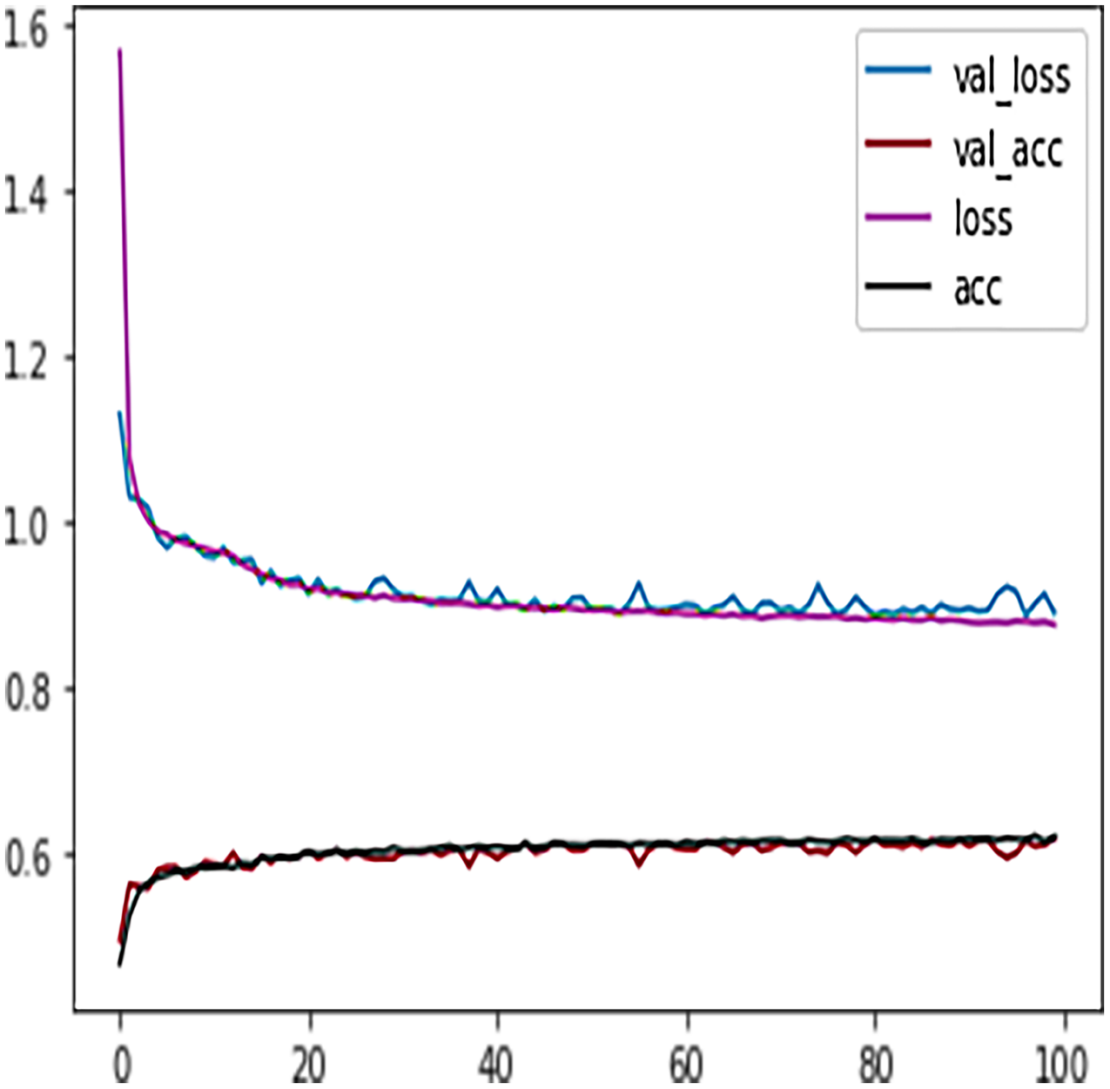
DFFNN training accuracy/loss, validation accuracy/loss.

Like DT, the prediction performance of DFFNN was evaluated first on four students’ performance categories, *i.e*., *Withdrawn, Fail, Pass, Distinction*. Subsequently, the prediction performance was evaluated on two categories, namely *Complete* and *Incomplete*. For four output classes, the DFFNN configuration was set to; activation function for hidden layer = Relu, input shape = 50, activation function for output layer = softmax, loss = categorical_crossentropy, optimizer = adam, kernel regularizer =mregularizers. A total of 12 metrics = accuracy, epochs = 100, batch size = 10. Figure 9 shows the validation loss-training loss and validation accuracy-training accuracy for DFFNN 100 epochs. It can be observed that both the validation loss and training loss have a similar pattern and decrease after each epoch. On the other hand, the validation accuracy and training accuracy is increasing with each successive epoch. Moreover, both the validation loss and validation accuracy are spreading away from training loss and training accuracy after epoch 60. This concludes that the model training process should be stopped after 60 epoch for accurate prediction, as the model performance is deteriorating.

#### DFFNN early stopping during the training process

The early stopping technique was used as our DFFNN model was trained too much. In this technique, the validation loss for the model is tracked, and once it begins increasing too much, the model training process is stopped. The early stopping Python class was imported from the TensorFlow Keras library with the following configurations.

$\rm {monitor}=`` val\_loss ",\; mode= `` min ", \; verbose=1,\; patience=10$

**Figure 10:**
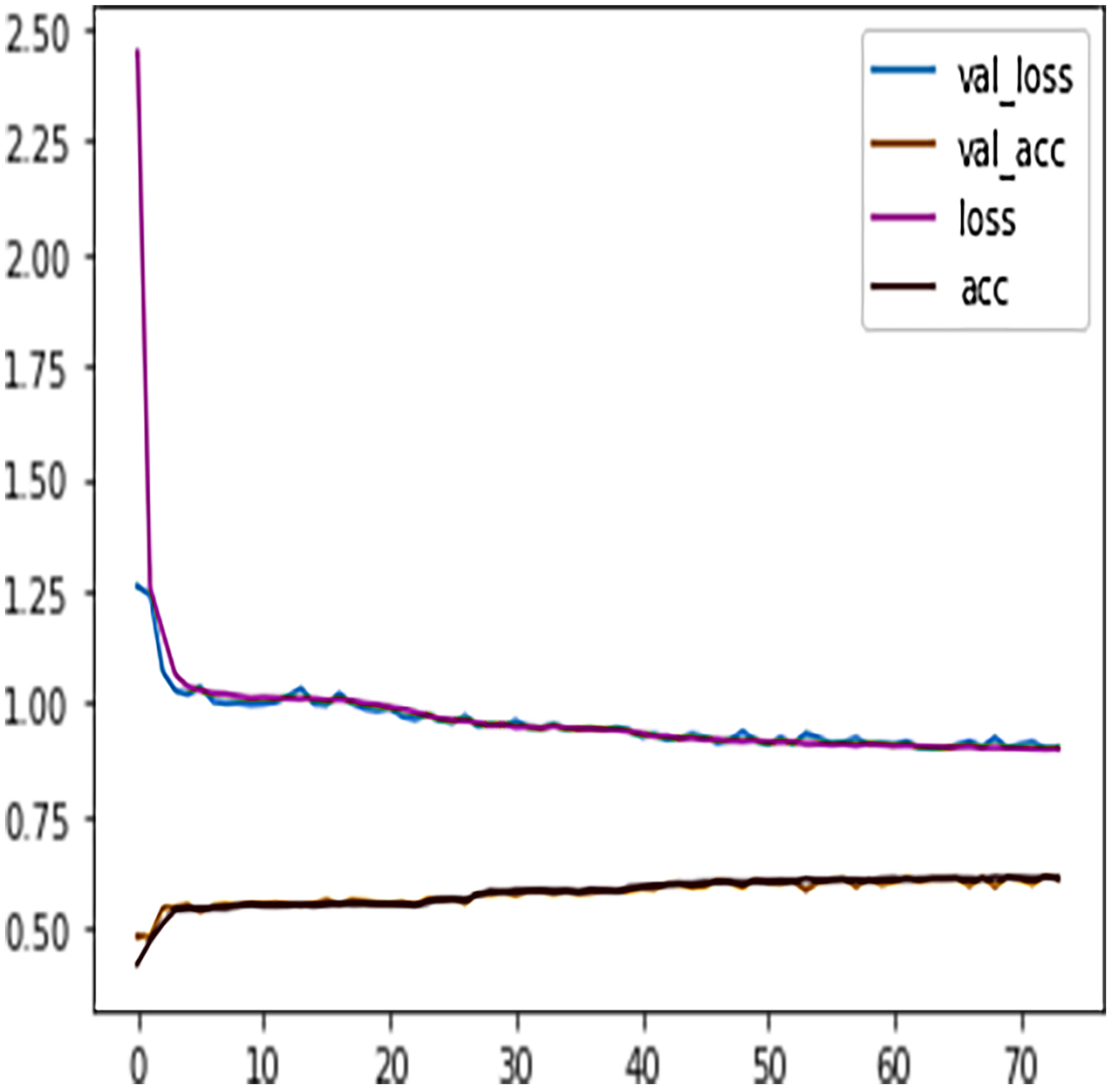
DFFNN early stopping during the training process.

We observed that at epoch 74, the model training process stopped as the model validation loss was increasing. Figure 10 shows the model early stopping scenario, where it can be observed that the validation loss was growing and spreading away from training loss after epoch 74. Thus, the early stopping technique helps avoid DL algorithms consuming more resources and overfitting the models.

#### Increasing DFFNN performance by dropping inactive neurons

**Figure 11:**
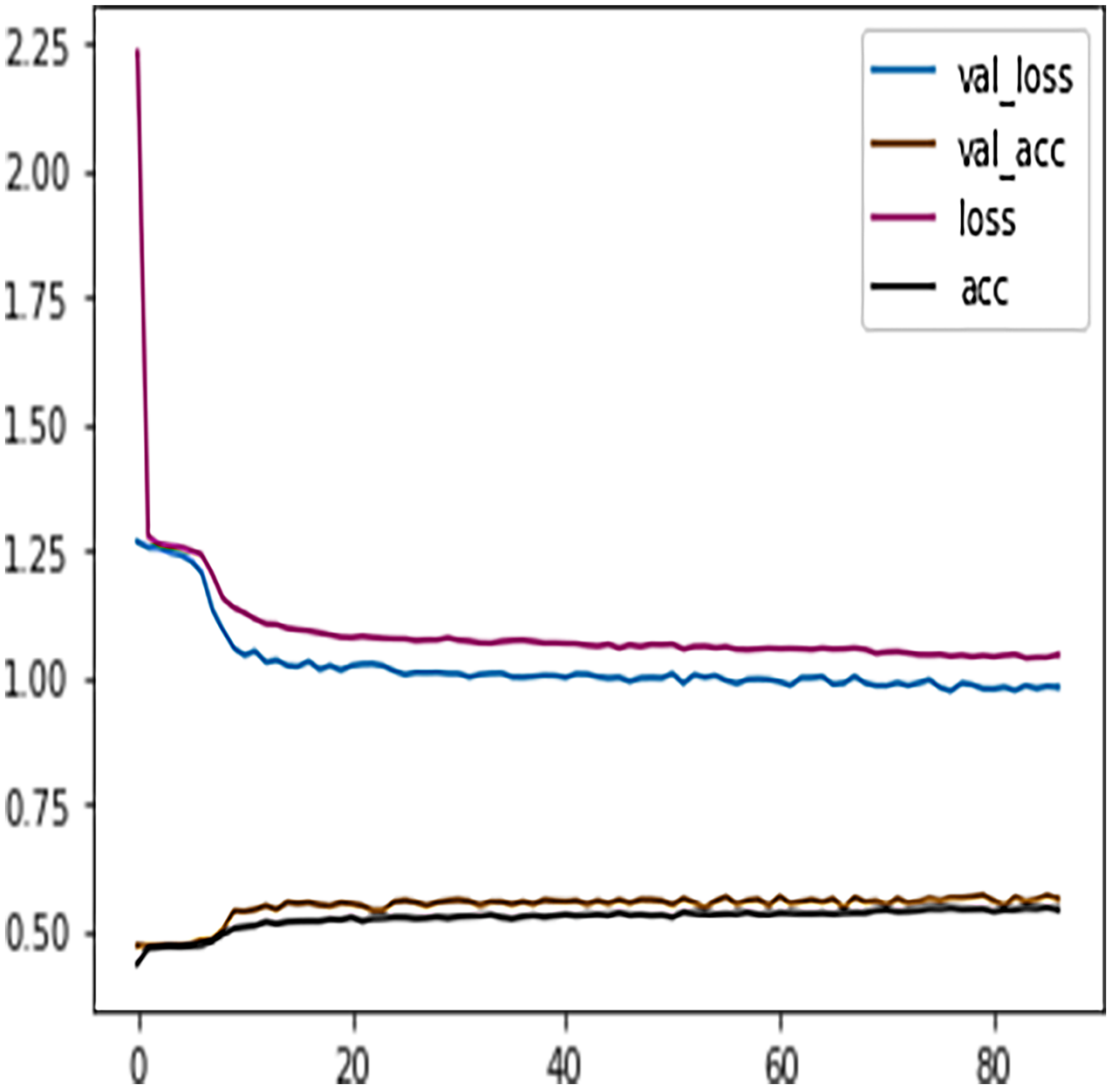
DFFNN early stopping after inactive neurons dropouts.

The DFFNN model’s performance can further be increased by adding a dropout layer that drops a percentage of inactive neurons and thus prevents the model from overfitting. The inactive neurons are those neurons that do not participate in the DFFNN training process. The Python dropout class was imported from the Keras layers package, and the dropout calls with a value of 0.5 were added to each dense layer. Suppose a neuron is not activated during the training process. In that case, the value of 0.5 indicates a probability of 50% that the dropout calls will deactivate the neuron. The DFFNN model was once again fitted and executed with the same parameters as the early stopping technique. This time the model stopped training at epoch 87, and from Fig. 11 we noticed that the model showed even better behavior with validation and learning loss essentially flattening out at the same rate.

Table 6 presents the precision, recall, f1 score, support, accuracy, macro average, and weighted average of the DFFNN model when trained on a multiclass problem. We noticed that the Pass grade received the highest values for precision, recall, and f1 score, whereas the model’s accuracy was 62%.

**Table 6:** DFFNN performance score for distinction, fail, pass and withdrawn grades.

DFFNN performance score	Precision	Recall	f1-score	Support
D	0.57	0.51	0.54	471
F	0.50	0.44	0.47	946
P	0.67	0.83	0.74	2,036
W	0.59	0.38	0.46	888
Accuracy			**0.62**	4,341
Macro avg	0.58	0.54	0.55	4,341
Weighted avg	0.61	0.62	0.60	4,341

**Note:**

Bold value is the accuracy score of the DFFNN model for distinction, fail, pass and withdrawn grades

#### Feature engineering: merging grades to increase DFFNN performance

**Figure 12:**
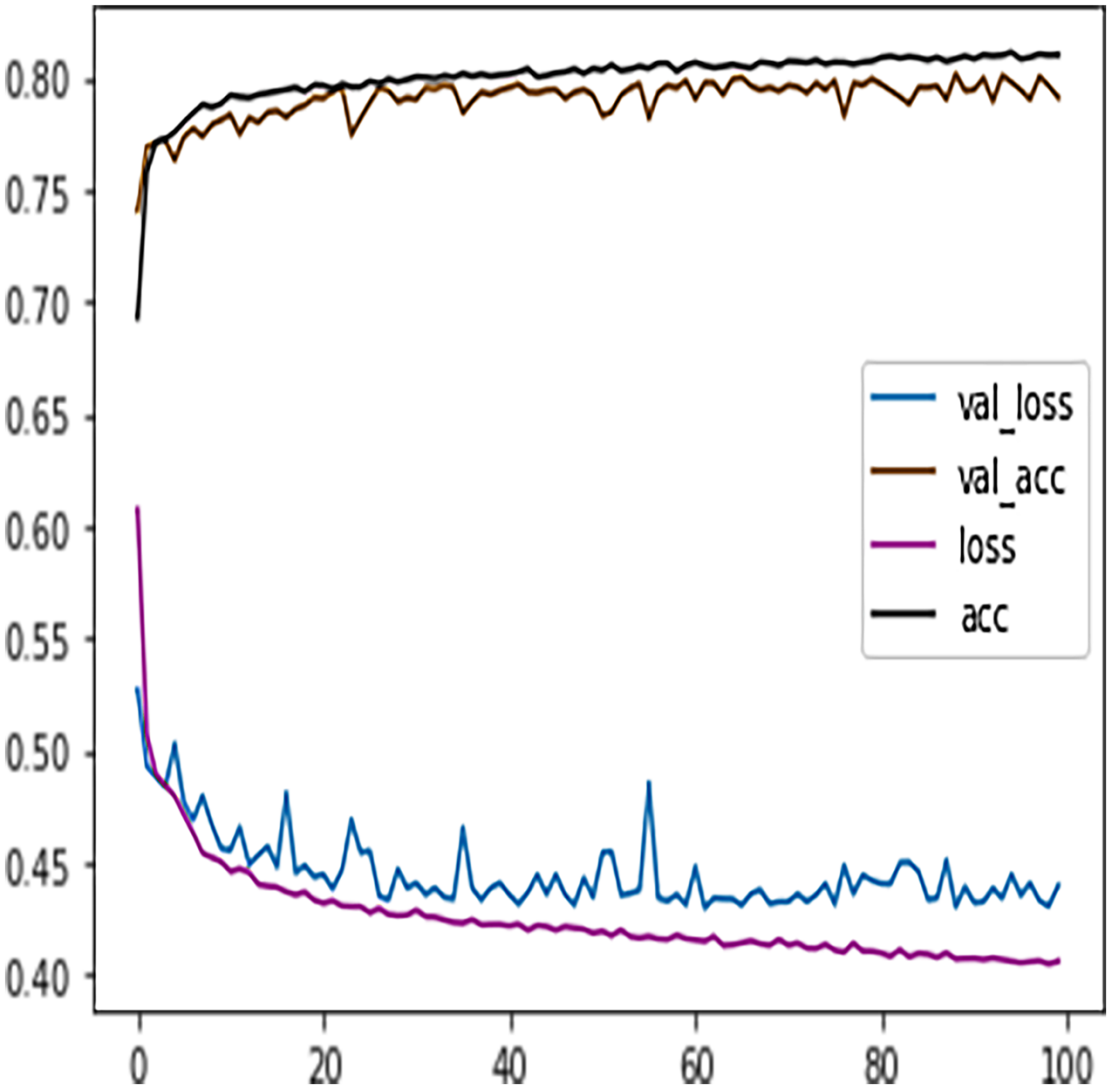
DFFNN training history for complete/incomplete grades.

**Figure 13:**
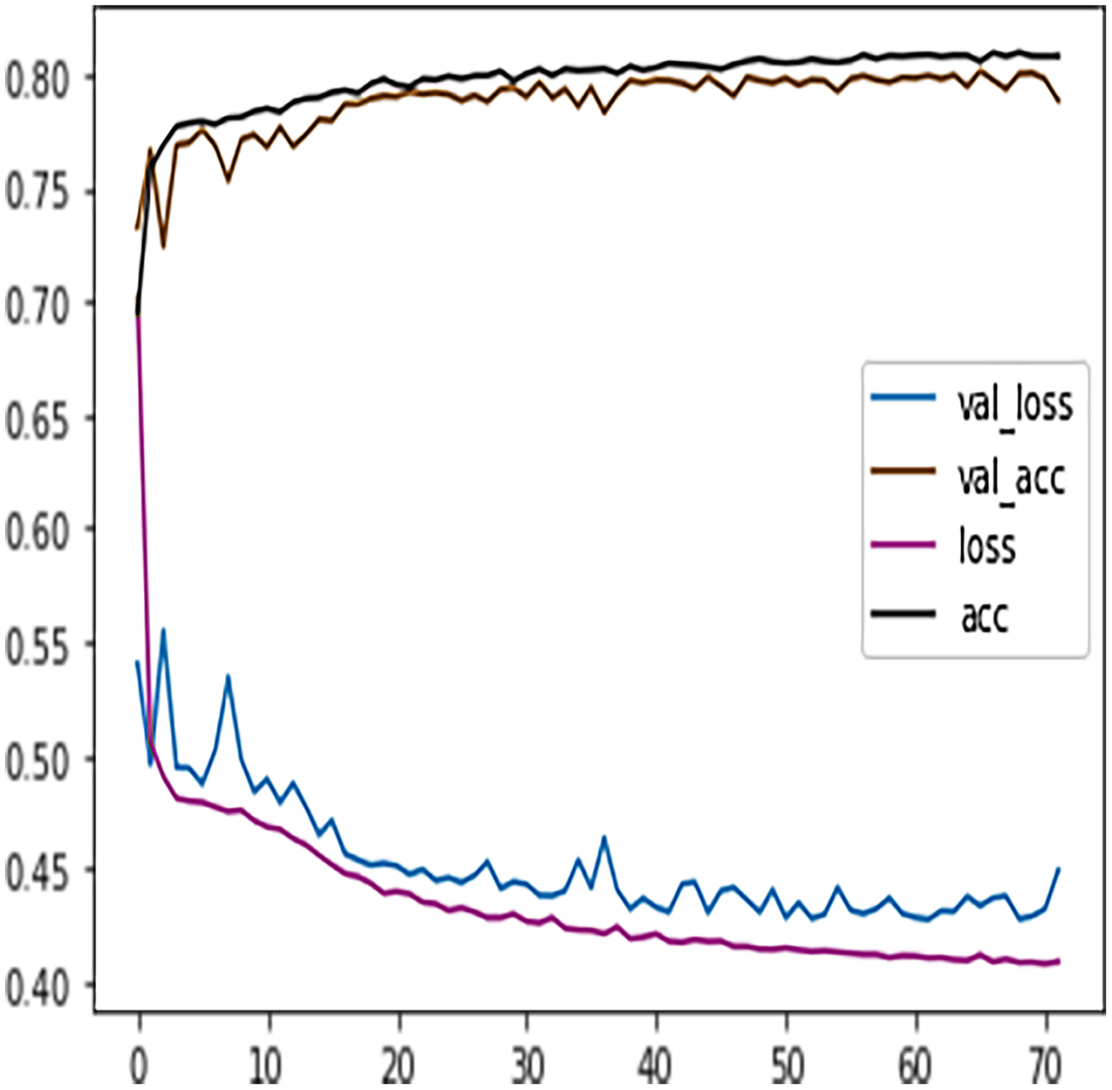
DFFNN early stopping for complete/incomplete grades.

**Figure 14:**
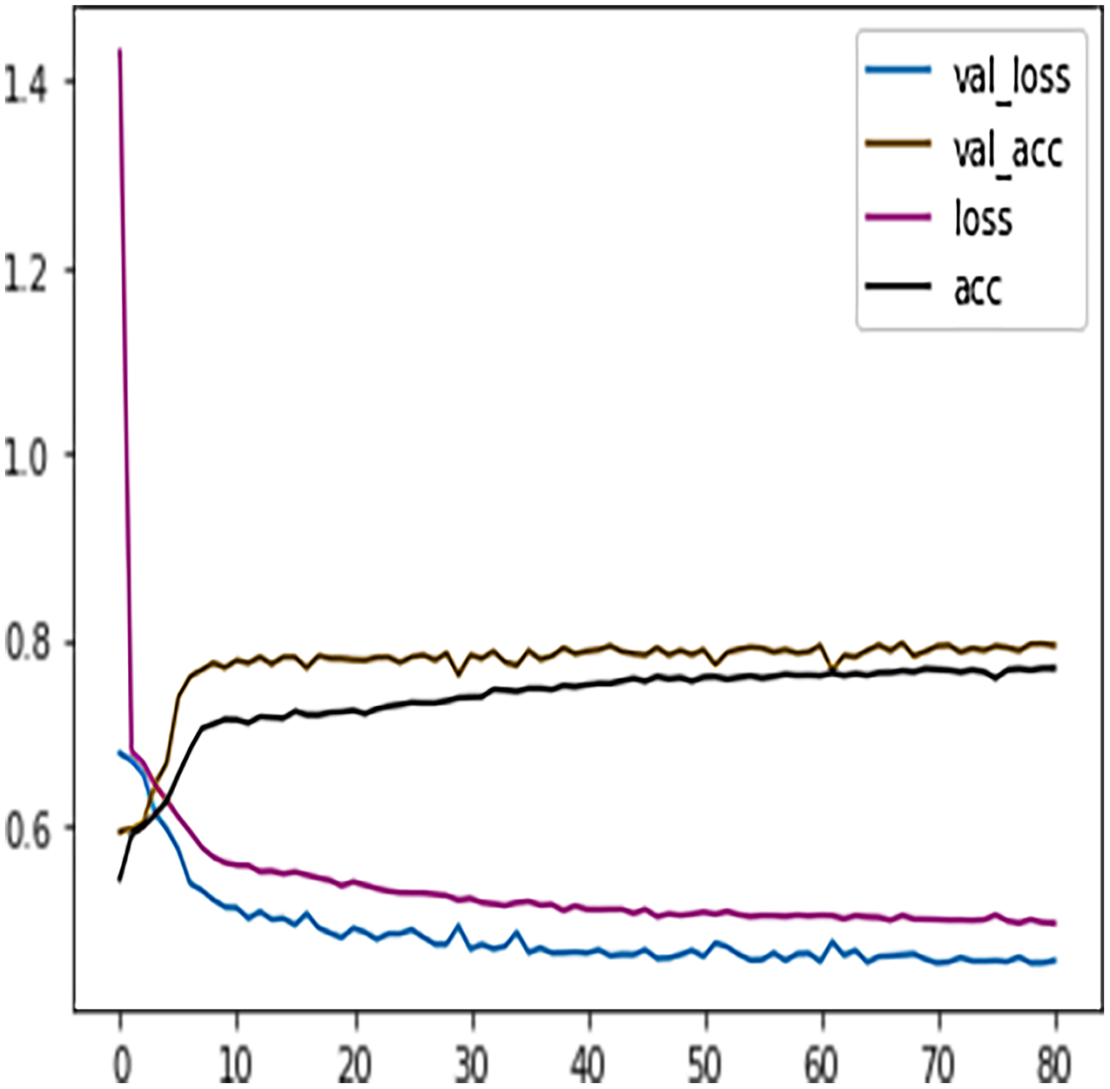
DFFNN early stopping and dropping inactive neurons for complete/incomplete grades.

Table 7 shows the DFFNN model precision, recall, f1-score, support, accuracy, macro average, and the weighted average for binary classification configuration. After the training process, the DFFNN model achieved a prediction accuracy of 79%, which is 18% more than the model trained for multiclass classification (61%). Figure 12 shows the DFFNN model training history. In each elapsing epoch, the training accuracy and validation accuracy increases, while the training loss and validation loss are decreasing. It can be only noticed that the validation loss is increasing and is spreading away from training loss at the end of the training process, which may cause the DFFNN model to overfit. Similar to the multiclass classification model, we used early stopping and layer dropout techniques to avoid the DFFNN model from suffering from overfitting and consume more resources. In Fig. 13, it can be noticed that after epoch 72, the validation loss was spreading away from training loss; therefore, the DFFNN training process was stopped by the early stopping technique. Similarly, in Fig. 14, it can be observed that the DFFNN training process was stopped at epoch 81 as the model did not experience any change in the training accuracy, training loss, validation accuracy, and validation loss.

**Table 7:** After feature engineering, DFFNN performance for complete/incomplete grades.

DFFNN performance score for complete/incomplete grades	Precision	Recall	f1-score	Support
Complete	0.78	0.90	0.83	2,526
Incomplete	0.82	0.64	0.72	1,815
Accuracy			**0.79**	4,341
Macro avg	0.80	0.77	0.78	4,341
Weighted avg	0.80	0.79	0.79	4,341

**Note:**

Bold is the accuracy score of the DFFNN model for complete and incomplete grades

**Figure 15:**
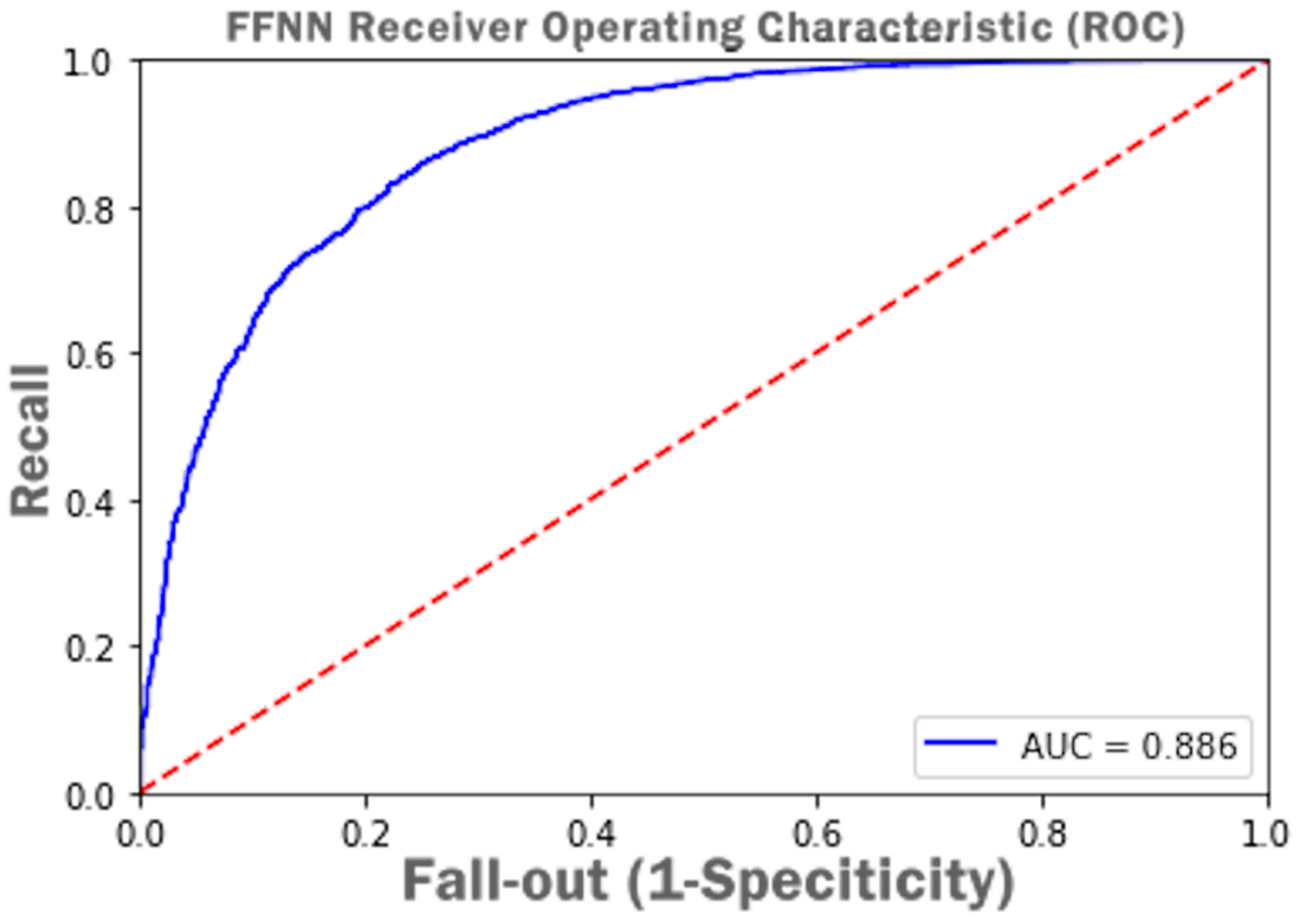
FFNN receiver operating characteristic (ROC) with AUC score.

Figure 15 presents Receiver Operating Characteristic (ROC) *i.e.*, probability curve and the value of Area Under Curve (AUC) as 0.886 which infer that the FFNN model accuracy of distinguishing between complete and incomplete class is 88%.

## Discussion and Limitations

We selected 11 ML and 1 DL algorithms to train the learner model and predict which one gives the best results. Gradient boosting, extra tree classifier, random forest’ criterion = ‘gini’, and random forest’ criterion = ‘entropy’ classifiers give the highest performance scores. However, we selected DT and FFNN models for performance improvement, as one of the objectives of this study is to improve models performance in an environment where they show unsatisfactory performance results.

Initially, when the data was fitted to the DT model, it generated unsatisfactory results with accuracy = 0.518, and f1 = 0.517. We noted that one of the reasons for the low-performance score was the class imbalance problem. To improve the DT model performance, feature engineering technique was performed, which resulted in merging Withdrawn and Fail classes to Incomplete class and Pass and Distinction classes to Complete class. When trained to predict the Incomplete and Complete class, the DT model performance improved with accuracy = 0.753 and f1 = 0.753 score. The Grid Search Cross-Validation (GSCV) technique further enhanced the DT model’s performance, increasing the DT model accuracy score upto 0.83. To further improve the DT model performance, AdaBoost and XGBoost ensembling techniques were used. After fitting the training and testing data to the AdaBoost and XGBoost classifier, an accuracy of 0.792 and f1 score of 0.791 was achieved. We noted that no improvement was made to the DT model after using the AdaBoost technique. Next, we fitted the data to the XGBoost classifier and achieved an accuracy of 0.82 and an f1 score of 0.81. Moreover, the XGBoost classifier was also used to determine the independent feature weights in predicting students’ final performance. In the last stage of the XGBoost model tuning process, early stopping technique was used to avoid underfitting and overfitting problems.

Similar to the DT model, the performance of FFNN was evaluated first on four students’ classes, namely, Withdrawn, Fail, Pass, Distinction. To avoid the FFNN model from generating inaccurate results, the model training process was stopped at 60 epoch. The early stopping technique helped the FFNN model to avoid consuming more resources and avoid overfitting problem. To further improve the FFNN model’s performance, inactive neurons were dropped from the neural network, thus assisting the model in preventing the overfitting problem. Like the DT model, we performed a feature engineering process to improve the FFNN model’s performance further. The withdrawn-fail grades were merged into Incomplete grade and pass-distinction grades to complete grade. When trained after the feature engineering process, FFNN achieved a performance accuracy of 79%, which was 18% more than the model trained for multiclass classification (61%).

The limitations of this study include not modeling the students’ behavior at different stages of course length. Modeling students’ behavior at different stages of course length would help instructors in knowing about the weaknesses of students earlier in the course thus would have taken preemptive measures to avoid students from failure or dropouts. Moreover, only 1 DL model *i.e*., FFNN was used for modeling students’ behavior. In the future, other DL algorithms such as RNN, CNN, Transformers, LSTM, and deep Boltzmann machines would be used for students’ performance prediction and how various techniques can be used to improve DL models performance upon showing unsatisfactory results. Furthermore, the validity of the ML results may suffer from the following constraint in real-time.

- The models may suffer from poor transfer learning ability and integration.
- The models require structure training data and hand-crafted features.
- Every model may require a special training process and planning.
- The ML models may suffer from a slow learning process in real-time.

## Conclusion and Future Work

This study presents various ML models that can be used to predict students’ performance in VLE by considering the feature weights. In VLE, it is vital to decide which ML algorithm to choose for modeling students’ learning behavior as the right ML algorithm affects the online system’s performance. This study also presents how the ML model’s performance can be increased in a situation where the ML model is generating unsatisfactory results. As an example, we selected DT and DFFNN models for modeling students learning behavior in VLE and how their performance can be increased by using various techniques such as using grid search cross-validation, ensembling models, adaptive boosting, extreme gradient boosting, early stopping, feature engineering, and dropping inactive neurons from neural network hidden layers.

Out of 12 ML/DL models, we selected the DT and FFNN models to improve their performance as they were showing unsatisfactory performance results. Initially, the DT model showed a low accuracy = 0.518 and f1 = 0.517 score. To improve the DT model performance, a feature engineering technique was employed and subsequently higher accuracy = 0.753 and f1 = 0.753 score was achieved. Other performance improvement techniques such as Grid Search Cross-validation, ensembling, adaptive boosting, eXtreme Gradient Boosting (XGBoost), and early stopping were used and achieved an overall accuracy of 80.83% and Ff1 = 79.56. After training the FFNN model, initially, it achieved an accuracy of 62%. Similar to the DT model, a feature engineering technique was employed for the FFNN model, and it reached a performance accuracy of 79%. Other performance improvement techniques such as early stopping and dropping inactive neurons were also used to reduce the execution cost of the FFNN model.

Our research study can help instructors and educational institutes formulating proper guidelines to students by considering their feature weights, strengths, and weaknesses. In future work, we plan to deploy only deep learning algorithms such as Long Short-Term Memory (LSTM), Convolutional Neural Networks (CNNs), Self-Organizing Maps (SOMs), Recurrent Neural Networks (RNN), and Deep Artificial Neural Networks (DANN) to model students learning behavior in online settings and compare their performance results.

## Supplemental Information

10.7717/peerj-cs.803/supp-1 Supplemental Information 1 Students online activities count during VLE interaction.

10.7717/peerj-cs.803/supp-2 Supplemental Information 2 Assessments score relationship with the students final result.

10.7717/peerj-cs.803/supp-3 Supplemental Information 3 Gender wise final result of students with VLE clickstreams.

10.7717/peerj-cs.803/supp-4 Supplemental Information 4 Confusion matrix for the DT model.

10.7717/peerj-cs.803/supp-5 Supplemental Information 5 Merging VLE tables for the ML models input vectors.

10.7717/peerj-cs.803/supp-6 Supplemental Information 6 DFFNN confusion matrix for distinction, fail, pass, withdrawn grades.

10.7717/peerj-cs.803/supp-7 Supplemental Information 7 Gender wise prior education and assessments relationship.

## Additional Information and Declarations

### Data Availability

The following information was supplied regarding data availability: The data is available at https://analyse.kmi.open.ac.uk/open_dataset. The code of the model is available at Kaggle: https://www.kaggle.com/adnankust/utilizing-grid-search-cross-validation.
