# Supplementary figures and images for: Utilizing grid search cross-validation with adaptive boosting for augmenting performance of machine learning models
Source: PeerJ Comput Sci. 2022 Feb 21;8:e803. doi: 10.7717/peerj-cs.803 (PMC9044349; doi:10.7717/peerj-cs.803)

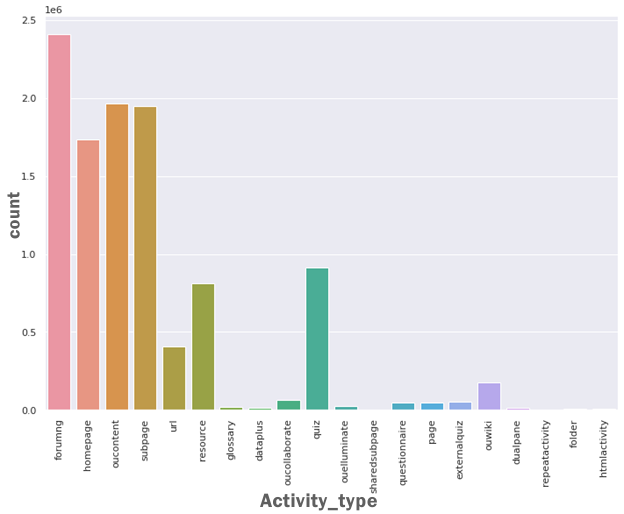

Supplement: Supplemental Information 1 [file peerj-cs-08-803-s001.png]

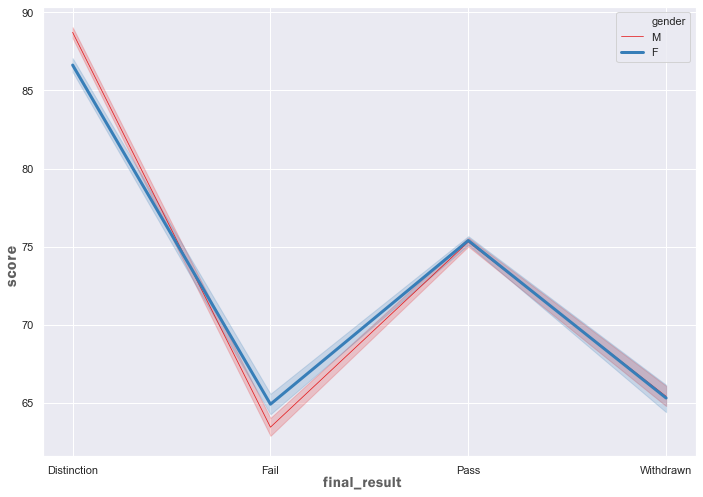

Supplement: Supplemental Information 2 [file peerj-cs-08-803-s002.png]

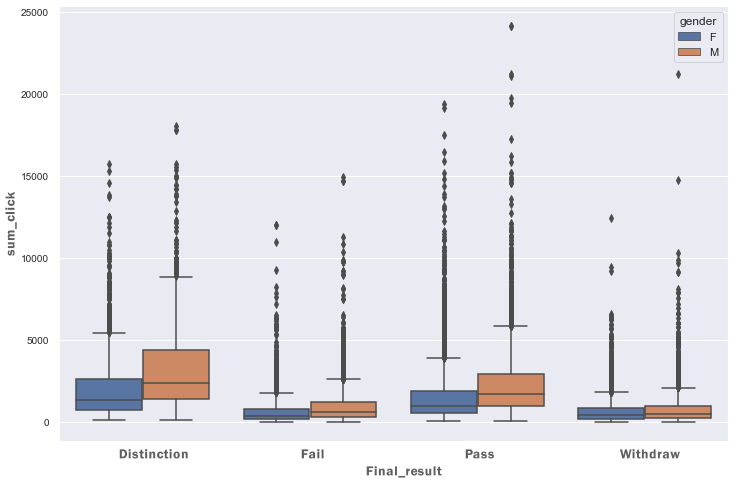

Supplement: Supplemental Information 3 [file peerj-cs-08-803-s003.png]

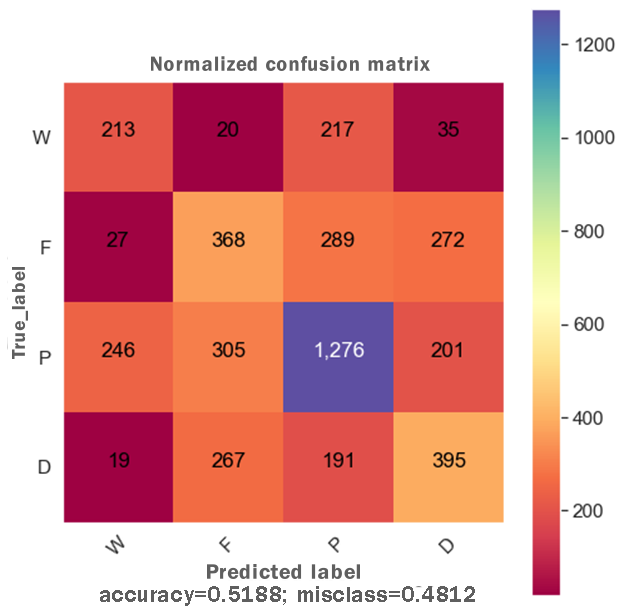

Supplement: Supplemental Information 4 [file peerj-cs-08-803-s004.png]

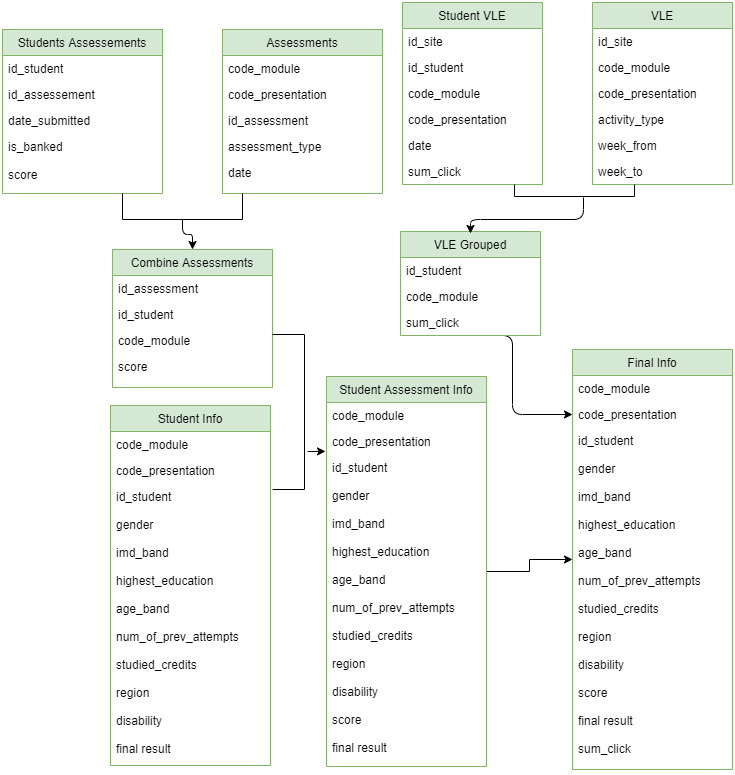

Supplement: Supplemental Information 5 [file peerj-cs-08-803-s005.png]

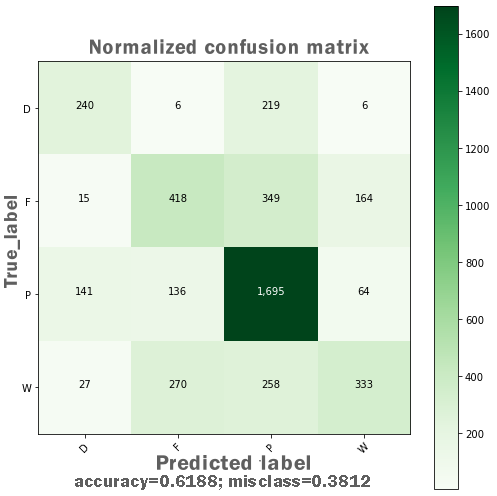

Supplement: Supplemental Information 6 [file peerj-cs-08-803-s006.png]

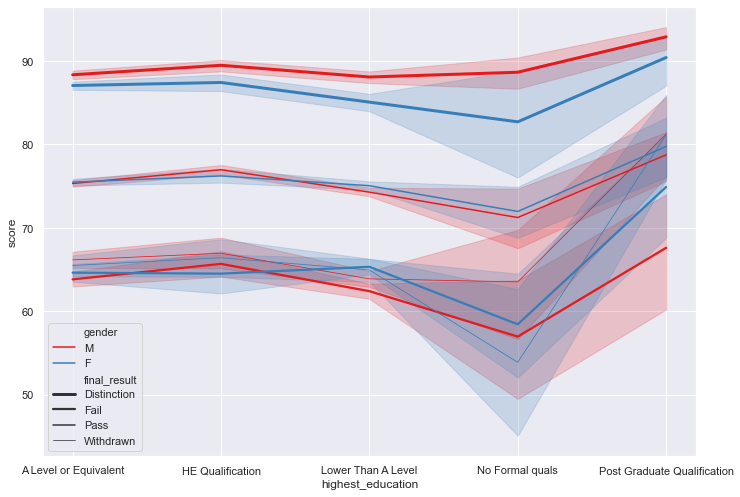

Supplement: Supplemental Information 7 [file peerj-cs-08-803-s007.png]
